# Endocytosis gets in tune with action potential bursts

**DOI:** 10.7554/eLife.01234

**Published:** 2013-08-20

**Authors:** Melissa A Herman, Christian Rosenmund

**Affiliations:** 1 **Melissa A Herman** is at Neurocure NWFZ, Charité Universitätsmedizin Berlin, Berlin, Germanymelissa.herman@charite.de; 2 **Christian Rosenmund** is an *eLife* reviewing editor, and is at Neurocure NWFZ, Charité Universitätsmedizin Berlin, Berlin, Germanychristian.rosenmund@charite.de

**Keywords:** endocytosis, synaptic vesicle, dynamin, phosphorylation, Mouse, Rat

## Abstract

Neurons use a calcium-dependent mechanism to optimize the rate at which synaptic vesicles are recycled.

**Related research article** Armbruster M, Messa M, Ferguson SM, De Camilli P, Ryan TA. 2013. Dynamin phosphorylation controls optimization of endocytosis for brief action potential bursts. *eLife* **2**:e00845. doi: 10.7554/eLife.00845

**Image** The rate of vesicle recycling via endocytosis varies with the number of action potentials

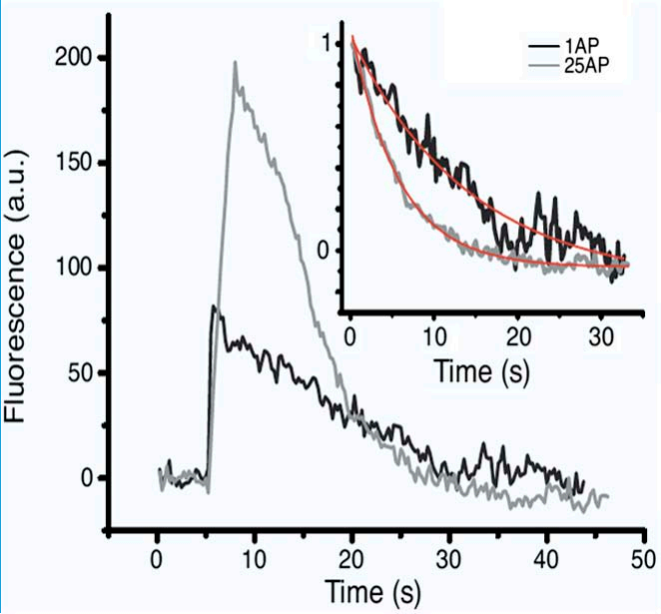

The arrival of an action potential at a synapse connecting two neurons causes calcium channels in the presynaptic membrane to open, and the resulting influx of calcium triggers a sequence of events that ends with the regeneration of the action potential in the postsynaptic cell. First, vesicles filled with neurotransmitter fuse with the membrane of the presynaptic neuron and release their contents into the synapse. These neurotransmitter molecules can then bind to receptors on the postsynaptic cell, enabling the action potential to be regenerated. However, the presynaptic neuron must also recycle those vesicles that have been released to ensure that the whole process can be repeated when the next action potential arrives.

It is tempting to hypothesize that the release of the vesicles (a process known as exocytosis) and their recycling (endocytosis) are initiated or controlled by common signalling mechanisms. It is known that exocytosis is triggered by an influx of calcium ions, but the relationship between calcium ions and endocytosis is somewhat tenuous (for review, see Yamashita, 2012). Now in *eLife*, Tim Ryan, Pietro De Camilli, and co-workers—including Moritz Armbruster as first author—report that calcium entry during bursts of action potentials has an important role in optimizing the rate of endocytosis (Armbruster et al., 2013).

Previous studies have found that an increase in calcium influx has a variety of effects on the rate of endocytosis, ranging from inhibition (von Gersdorff and Mathews, 1994) to acceleration (Sankaranarayanan and Ryan, 2001; Wu et al., 2009). Armbruster and co-workers—who are at Cornell University and Yale University—have now reconciled these results by carefully examining how the rate of endocytosis varies as a function of the number of action potentials delivered at a given frequency, referred to as a burst. They revealed that endocytosis slows down as the number of action potentials increases from 25 to 100, and that this deceleration requires calcium. This is in contrast to previous work, which found the rate of endocytosis to be constant over this range (Balaji et al., 2008). However, Armbruster et al. note that this previous study compared the ensemble average rate between boutons, and may thus have been less sensitive than their own study, which examined the rate of endocytosis within a bouton following differing numbers of action potentials.

Perhaps the most surprising finding came when Armbruster et al. examined the effect of a single action potential on the rate of endocytosis. Given that endocytosis slows as the number of action potentials increases from 25 to 100, they expected it to occur rapidly in response to a single action potential. On the contrary, they found that endocytosis was slow after a single spike, and accelerated as the number of action potentials increased to 25. It seems, therefore, that the rate of endocytosis shows a biphasic relation to the number of action potentials, with the fastest rate corresponding to an intermediate stimulation range (Figure 1A).

**Figure 1.**
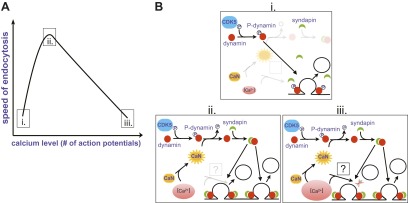
Changes in the speed of endocytosis and the underlying signalling pathways. (**A**) Schematic representation of the relationship between the speed of endocytosis, the level of intracellular calcium produced by increasing numbers of action potentials, and the signalling pathways activated by intracellular calcium when there is just a single action potential (i), a small burst of action potentials (ii), and a large burst of action potentials (iii). (**B**) Calcium levels are low when there is just one action potential (i), so calcineurin (CaN) is not activated, allowing Cdk5 to maintain high levels of phosphorylation of dynamin (P-dynamin). As the number of action potentials increases (ii), there are more calcium ions to activate the calcineurin, which results in a shift towards dephosphorylated dynamin. This can then bind to syndapin, and this interaction increases the rate of endocytosis. However, as the number of action potentials, and hence calcium levels, increase further (iii), a second calcium-dependent pathway with unknown molecular components is activated, and this reduces the rate of endocytosis.

The next question was obvious: what is the mechanism behind this modulation of endocytosis by the number of action potentials? Given that the process clearly involved calcium, Armbruster et al. turned to proteins involved in endocytosis that are also substrates for calcineurin, an enzyme activated by calcium ions. This enzyme, which removes phosphate groups, and its counterplayer Cdk5 (which adds phosphate groups) have been implicated in the dynamic regulation of synaptic strength (Kim and Ryan, 2013).

One of the targets of calcineurin/Cdk5 is dynamin, a small GTPase enzyme that helps newly formed vesicles to bud off from the presynaptic membrane during endocytosis (Ferguson and De Camilli, 2012). In an elegant set of rescue experiments, Armbruster et al. showed that the acceleration of endocytosis can only occur if phosphate groups have been removed from two previously identified sites on dynamin. Since dephosphorylated dynamin interacts with another protein called syndapin, which is also involved in endocytosis, they propose the following signalling pathway: The firing of multiple action potentials leads to a brief elevation of presynaptic calcium (Figure 1B (i)); this calcium activates calcineurin, which then dephosphorylates dynamin at two sites (ii); and a dynamin-syndapin complex then accelerates endocytosis (iii). Exactly how this last step occurs is unclear, although one possibility is that syndapin accelerates the recruitment of dynamin to sites of endocytosis (Koch et al., 2011).

Intriguingly, previous studies suggest that stimulus-induced dephosphorylation of dynamin lasts approximately 40 s (Robinson et al., 1994), which is also roughly the length of time for which endocytosis acceleration persisted after a small burst (Armbruster et al., 2013). This is the strongest piece of evidence that calcium regulates the speed of endocytosis by modulating the balance of calcineurin/Cdk5 activity. Whether dynamin is the only target of this pathway remains to be determined.

The mechanism responsible for the slowing of endocytosis with larger numbers of action potentials remains unclear (Figure 1), but it is not related to phosphorylation of dynamin at the syndapin interaction site. This suggests that the recycling of vesicles might depend on at least two separate calcium-dependent processes. One possibility is that endocytosis slows as the number of action potentials increases because the accumulation of exocytic proteins on the surface of the presynaptic cell leads to saturation of endocytic machinery, as previously described (Balaji et al., 2008). Moreover, because endocytosis still occurred in experiments where the dynamin phosphorylation state could not be altered, this pathway is likely not responsible for the calcium-dependent initiation of endocytosis (Gad et al., 1998).

Another intriguing aspect of these results is the potential for cell-type specific modulation. The acceleration of endocytosis with increasing action potential number in the small burst range could be regulated by a cell’s intrinsic calcium buffering capacity or its calcineurin/Cdk5 activity balance. Consistent with this, Armbruster et al. observed differences in the degree of regulation between cultures of neurons from different species and brain regions. Additionally, given that intrinsic firing patterns vary between cell types in vivo, it is possible that the standard burst size of a cell may define its baseline endocytosis rate. In conclusion, these findings add endocytosis to the list of dynamically regulated processes within synaptic transmission, which enable synapses to optimize information transfer between neurons in order to form networks within the brain.
